# Back to basics: measuring the impact of interventions to limit the spread of COVID-19 in Europe

**DOI:** 10.1186/s13690-022-00830-5

**Published:** 2022-03-09

**Authors:** Dominic Cortis, Fiona Vella King

**Affiliations:** Department of Insurance, Faculty of Economics, Management and Finance, University of Malta, Msida, Malta

**Keywords:** Non-pharmaceutical interventions, COVID-19, Europe, Lockdown effectiveness

## Abstract

**Background:**

Following the emergence of the COVID-19 pandemic in Europe at the start of 2020, most countries had implemented various measures in an attempt to control the spread of the virus. This study analyses the main non-pharmaceutical interventions and their impact on the rate by which cumulative cases and deaths were growing in Europe during the first wave of this pandemic.

**Methods:**

The interventions analysed are the school closures, restrictions on travel, cancellation of events, restrictions on gatherings, partial and full lockdowns. Data was collected on the implementation date of these interventions, and the number of daily cases and deaths during the first wave of the pandemic for every country and territory geographically located in Europe. The study uses growth rates to calculate the increase in cumulative cases and deaths in Europe before, during, and after these interventions were implemented.

**Results:**

The results show that decisions to close schools, cancel events, and restrict travel were taken during the same time period, whereas the decisions for the other interventions were taken when the growth rates were similar. The most effective interventions at lowering the rate by which cumulative cases were increasing were the travel restrictions, school closures, and the partial lockdown, while most effective against cumulative deaths were the partial lockdown, travel restrictions, and full lockdown.

**Conclusion:**

All the interventions reduced the rate by which cumulative cases and deaths were increasing with the partial lockdowns being the most effective from the other interventions, during the first wave of the pandemic in Europe.

## Background

The COVID-19 pandemic has been the focus of interest for the past months and will continue to serve as a platform for discussions in health management for many years. Typical study cases include an understanding of differences in frequency and severity (for example [6, 15, 16]; and [35]), its effect on other aspects (for example [24, 27, 28]) as well as measures to contain the epidemic (for example [2, 8, 15]).

This paper analyses the effects of the main six non-pharmaceutical interventions (NPIs) and their impact on the rate by which cumulative cases and deaths were increasing during the first wave of the pandemic in Europe. Non-pharmaceutical interventions are defined as measures that can be taken to limit the spread of a virus. These measures need to be taken by governments to reduce transmission in the absence of pharmaceutical interventions such as vaccines [13].

## Method

Our investigation focuses on the first wave in Europe^1^ which started at the end of January 2020 when the first cases were being reported, and ended around the end of May when the total number of daily cases for each country in the study summed up together remained below 20,000 and many countries had begun easing the restrictions implemented in the previous months [29].

### Data collection

Data was collected on the six NPIs listed in Table 1, and the date on which they were implemented during the first wave of the pandemic in Europe.

**Table 1:** List of Non-Pharmaceutical Interventions and their description

Non-Pharmaceutical Intervention	Description
**1.** Closure of Schools	Closure of all schools and universities
**2.** Travel Restrictions^a^	Closure of land and/or air borders
**3.** Cancellation of Public Events^b^	Required cancellations of all large^c^ events
**4.** Restrictions on Public Gatherings	Limiting outdoor and/or indoor gatherings
**5.** Partial Lockdown	Required to not leave the house with exceptions for daily exercise, grocery shopping, and ‘essential’ trips
**6.** Full Lockdown	Required to not leave the house with minimal exceptions (e.g. allowed to leave only once every few days, or only one person can leave at a time, etc.)

^a^This study focuses on international travel restrictions

^b^We use the term ‘cancellation of public events’ and ‘cancellation of events’ interchangeably

^c^Events with groups of over 50 people were considered as large events

This data was collected for all countries and territories geographically located in Europe using various secondary sources of data (Appendix 1). For the majority of countries, the ACAPS^2^ global dataset was used. However, this dataset did not include data for Liechtenstein, Isle of Man, Gibraltar, Channel Islands, and Vatican City. The data for these countries was taken from each country’s respective government website on COVID-19 [10, 18, 19, 21, 31, 34].

Data was also collected on the number of daily cases and deaths from the European Centre for Disease Prevention and Control (ECDC) dataset on the geographic distribution of COVID-19 cases worldwide for all countries, except for the Vatican City for which data was taken from the Worldometer Coronavirus Statistics website [9, 40].

### Data analysis

The first part of the analysis involved identifying the order in which the interventions were implemented. Measures of central tendency including the median and mean were used to find the average number of days each NPI was implemented after the first intervention. The standard deviation was also calculated as it showed the variability in the data.

The analysis also involved the calculation of nine growth rates that represent the percentage increase in the number of cumulative cases and deaths for each NPI. It is estimated that the incubation period for COVID-19 is on average 5–6 days, but it could also take up to 14 days for symptoms to appear [23, 36, 39].

**Fig. 1:**
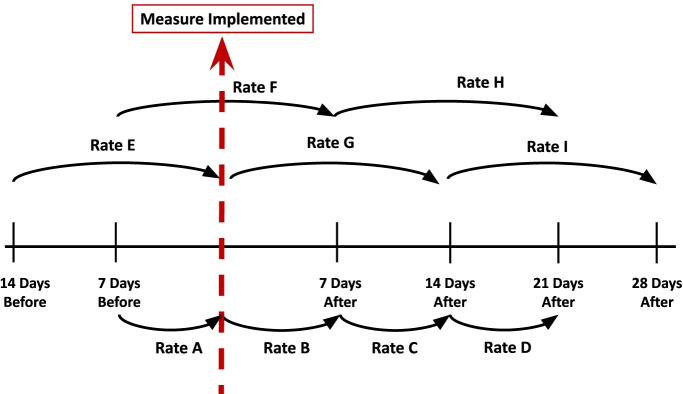
Rates of Growth

Therefore, this analysis used two sets of rates to analyse the effect of NPIs, as can be seen in Fig. 1. The first set of rates (A, B, C, D) show the percentage increase in seven-day periods, while the second set of rates (E, F, G, H, I) show the percentage increase in fourteen-day periods.

Each rate of growth was calculated for every country individually and for each of the six NPIs. Measures of central tendency, including both the median and mean were then used to find the overall rate of growth for each rate and for each NPI. With respect to the mean, the geometric mean was chosen since it is typically used when dealing with growth rates [7].

## Results

### Order of implementation

Forty-six out of fifty-four countries and territories in Europe implemented the cancellation of events as one of their first measures to control transmission of the virus. Out of the remaining eight countries, five countries implemented school closure as their first NPI and three countries set international travel restrictions first. The countries that set international travel restrictions quickly followed their decision with school closures prior to cancelling large events (Appendix 4).

Table 2 shows the order in which the other five NPIs were implemented.

**Table 2:** Number of Days between implementation of the cancellation of events NPI and the other five NPIs

	School Closures	Travel Restrictions	Restrictions on Gatherings	Partial Lockdown	Full Lockdown
**Median**	1.00	5.00	1.00	7.00	24.00
**Mean**	1.98	4.92	4.75	8.45	24.29
**Standard Deviation**	5.71	6.72	6.45	6.44	15.24

School closures were implemented on average between 1 and 2 days after events were cancelled. This was followed by the restrictions on gatherings and travel which were implemented on average within less than a week after events were cancelled. The lockdowns were the last decisions taken, with the partial lockdown implemented on average over a week after events were cancelled, and the full lockdown implemented on average 24 days after events were cancelled. The standard deviation shows that this was not the case for all countries, since some took either much longer to implement additional restrictions or were quicker to do so.

### Cumulative cases

Table 3 summarises the median growth rates in terms of cumulative cases for each growth rate and every NPI, with the mean growth rates also giving similar results (Appendix 2). Rates A and E measure the growth rate before the NPI was implemented, while Rate F measures the growth rate during the implementation of the NPI. All other rates measure the growth rate after the NPI was implemented.

**Table 3:** Median Growth Rates in terms of Cumulative Cases

Median Rate	School Closures	Travel Restrictions	Cancellation of Events	Restrictions on Gatherings	Partial Lockdown	Full Lockdown
**A**	528%	509%	621%	394%	406%	147%
**B**	483%	281%	629%	317%	195%	65%
**C**	155%	125%	239%	133%	105%	26%
**D**	77%	66%	99%	70%	48%	21%
**E**	4264%	3546%	2533%	3050%	3461%	700%
**F**	2773%	2580%	3481%	2477%	1487%	307%
**G**	1567%	860%	2060%	925%	555%	110%
**H**	336%	276%	550%	274%	221%	56%
**I**	155%	122%	225%	119%	83%	39%

As a first example, we compare the partial lockdown median cumulative rates. The figures for rate E and G are 3461% and 555% respectively. This means that if there were 100 cases 2 weeks prior to school closures, there would have been 3561 cases by the day partial lockdowns were effective and 23,324 cumulative cases to date 2 weeks later. The typical growth was 35-fold in the 2 weeks up to the NPI but ‘only’ six-fold 2 weeks later.

Similarly, we can clarify rates A, B and C for travel restrictions. If there were 100 cumulative cases a week before travel restrictions is implemented, there were typically 609 (+ 509%) cases on that day, 2320 (+ 281%) cases a week later and 5221 (+125%) cases a week after that (2 weeks after decision is taken). While the absolute increase is larger as time passes, the relative change is lower. In the week just prior to the decision, there was a six-fold increase (100 to 609) but the difference 2 weeks later was about double (2320 to 5221).

**Fig. 2:**
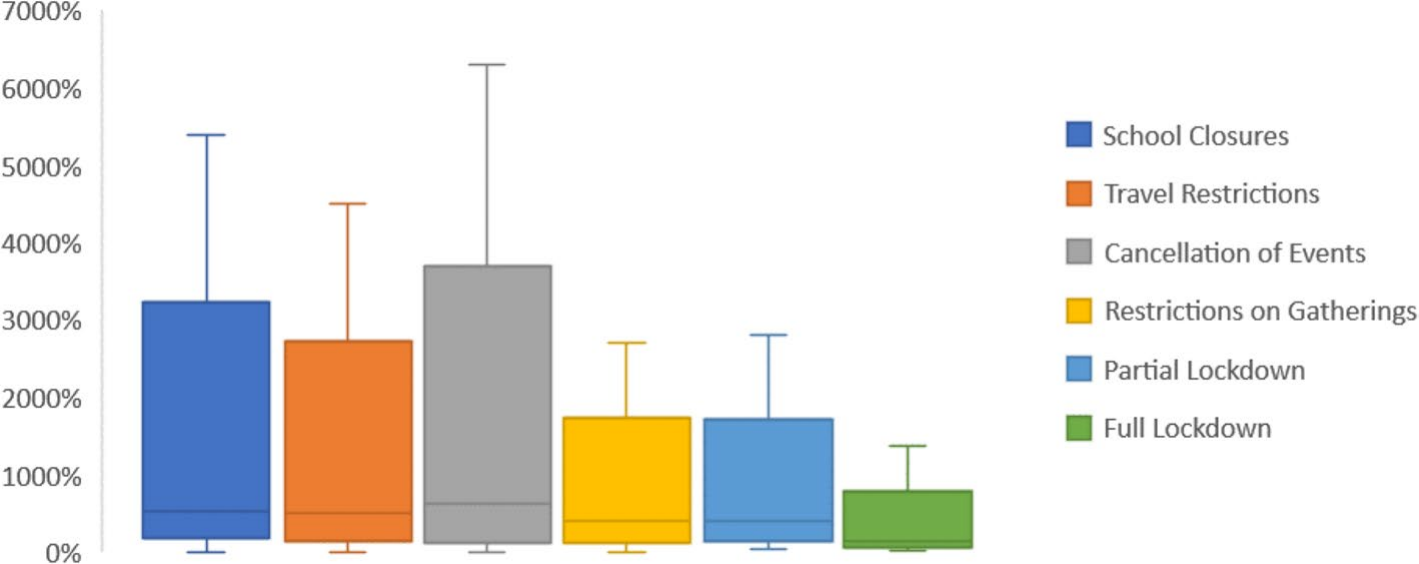
Rate A in terms of Cumulative Cases

Figure 2 shows the distribution of incident growth rates in the week preceding the decision of any NPI. The widest distribution of COVID-19 cases was occurring prior to the decision to close schools, restrict travel and cancel events. This can be explained by the fact that most countries took these three decisions during a similar timeframe despite being at different experience of the incidence growth rate.

#### Weekly rates (rates A – D)

Given the length of the incubation period, some of the individuals exposed a week before an intervention was implemented could have developed symptoms up to a week after. Therefore, Rate B is expected to be similar or smaller than Rate A, especially if other interventions have already been implemented. With the interventions in place for a longer time, Rate C and D should decrease further from Rate A. Table 4 shows the percentage change between the weekly median rates.

**Table 4:** Percentage change between weekly growth rates in terms of cumulative cases

% Change	School Closures	Travel Restrictions	Cancellation of Events	Restrictions on Gatherings	Partial Lockdown	Full Lockdown
**Rate A to Rate B**	−7%	− 37%	1%	− 16%	− 42%	−33%
**Rate A to Rate C**	− 59%	− 63%	−53%	− 53%	− 60%	− 49%
**Rate A to Rate D**	−72%	− 73%	−72%	− 66%	−71%	− 51%

We can again use the travel restrictions rate changes as an example. In the previous section we noted that in the week just prior to the decision, there was a six-fold increase (100 to 609) but the weekly-difference 2 weeks later was about double (2320 to 5221). This can be measured to be about a third of the old cumulative growth (since double is a third of a sixth). The exact measure is that the growth rate in the period C is 36.94%^3^ of the growth rate in period A which is a 63.05% difference.

Cumulative cases were increasing at the highest rate before events were cancelled. Rate A was also high for the school closures and travel restrictions NPIs. Before the other interventions were implemented the growth rate decreased slightly and decreased even further before the full lockdown.

The partial lockdown and travel restriction were two of the most effective interventions as they had the highest improvement from Rate A to both Rates B and C, lowering the growth rate by over 60%. Over another week, the travel restrictions had the highest improvement, followed by the school closures, cancellation of events, and partial lockdown, all of which lowered the growth rate by over 70%.

#### Fortnightly rates (rates E to I)

Due to the length of the incubation period, Rate F is expected to be similar to Rate E. With the interventions in place longer, Rates G, H, and I are expected to decrease from Rates E and F. Table 5 shows the percentage change between these median rates.

**Table 5:** Percentage change between fortnightly growth rates in terms of cumulative cases

% Change	School Closures	Travel Restrictions	Cancellation of Events	Restrictions on Gatherings	Partial Lockdown	Full Lockdown
**Rate E to Rate F**	−34%	− 27%	36%	− 18%	− 55%	−49%
**Rate E to Rate G**	− 62%	−74%	−18%	− 67%	− 82%	−74%
**Rate F to Rate H**	− 85%	− 86%	− 82%	− 85%	−80%	− 62%
**Rate E to Rate I**	−94%	− 94%	− 88%	− 93%	−95%	−83%

Cumulative cases were increasing at the highest rate from 2 weeks before schools were closed and travel was restricted. Rates E and F were slightly lower for the other NPIs, and even lower for the full lockdown, which means that 2 weeks before their implementation cumulative cases were increasing at a lower rate.

The partial lockdown was the most effective as it had one of the highest improvements between growth rates, followed by the school closures and travel restrictions which were also more effective in comparison to the other NPIs.

### Cumulative deaths

Table 6 summarises the median growth rates in terms of cumulative deaths for each growth rate and for each NPI, with the mean growth rates giving similar results (Appendix 3).

**Table 6:** Median Growth Rates in terms of Cumulative Deaths

Median Rate	School Closures	Travel Restrictions	Cancellation of Events	Restrictions on Gatherings	Partial Lockdown	Full Lockdown
**A**	567%	625%	335%	313%	505%	202%
**B**	642%	600%	441%	456%	445%	103%
**C**	330%	271%	343%	275%	245%	45%
**D**	167%	131%	229%	126%	103%	25%
**E**	6250%	4275%	7200%	3707%	4833%	900%
**F**	3447%	2900%	1975%	3150%	2977%	495%
**G**	2117%	1900%	2220%	1920%	1500%	160%
**H**	1099%	832%	1807%	683%	703%	83%
**I**	476%	320%	700%	367%	255%	45%

#### Weekly rates (rates A to D)

Due to the length of the incubation period, Rate B is expected to be similar to Rate A. With the interventions in place for a longer time Rate C and D should decrease further from Rate A. Table 7 shows the percentage change between the median rates.

**Table 7:** Percentage change between weekly growth rates in terms of cumulative deaths

% Change	School Closures	Travel Restrictions	Cancellation of Events	Restrictions on Gatherings	Partial Lockdown	Full Lockdown
**Rate A to Rate B**	11%	−3%	24%	35%	−10%	−33%
**Rate A to Rate C**	−36%	−49%	2%	−9%	−43%	−52%
**Rate A to Rate D**	−60%	− 68%	− 24%	−45%	−66%	−59%

Cumulative deaths were increasing at the highest rate before the implementation of travel restrictions. Rate A was also high before the implementation of school closures and partial lockdown. The travel restrictions and lockdowns were the most effective at lowering the rate by which cumulative deaths were increasing as they had the highest improvements between growth rates.

#### Fortnightly rates (rates E to I)

Due to the length of the incubation period, Rate F is expected to be similar to Rate E. With the interventions in place longer, Rates G, H, and I are expected to decrease from Rates E and F. Table 8 shows the percentage change between these median rates.

**Table 8:** Percentage change between fortnightly growth rates in terms of cumulative deaths

% Change	School Closures	Travel Restrictions	Cancellation of Events	Restrictions on Gatherings	Partial Lockdown	Full Lockdown
**Rate E to Rate F**	−44%	−31%	−72%	−15%	− 38%	− 40%
**Rate E to Rate G**	− 65%	−54%	−68%	− 47%	− 68%	−74%
**Rate F to Rate H**	−66%	− 69%	−8%	−76%	− 74%	− 69%
**Rate E to Rate I**	− 91%	− 90%	− 89%	− 88%	− 93%	−86%

Cumulative deaths were increasing at the highest rate before the implementation of the cancellation of events and closure of schools. Directly after implementation, the cancellation of events, school closures, and lockdowns were the most effective as they had the highest improvements from rate E to F.

After 2 weeks, the school closures and lockdowns were the most effective. As the interventions were in place for another week, the restrictions on gatherings had the highest improvement, followed by the partial lockdown. Over time, the partial lockdown, school closures, and travel restrictions were the most effective.

Only France had deaths 2 weeks prior to schools closing and travel being restricted, and so, there was no discrepancy observed in Rate E for these NPIs. Whereas before the other interventions were implemented cumulative deaths were increasing by varying rates.

## Discussion

The high variability in the pre- and mid-intervention rates A, E and F would show that interventions were implemented when countries where in different stages of the pandemic in terms of cumulative cases and/or deaths. However, a low variability in these rates would mean that the interventions were implemented when countries reached the same or a similar stage of the pandemic and not necessarily during the same time period.

### School closures

The decision to close schools was taken by Europe during the same time period since cumulative cases were increasing by varying rates between countries. The discrepancy observed in terms of cumulative deaths showed that this time period was the start of the pandemic. In fact, 2 weeks before implementation, only France had recorded any deaths and by the following week deaths started increasing by low and similar rates in more countries [33].

The results obtained coincide with the claims made by Brauner et al. [3], that the closure of schools and universities does reduce transmission of COVID-19. When comparing growth rates, the closure of schools was observed to be one of the most effective at improving the rate by which cumulative cases were increasing over time. On the other hand, even though it was effective at lowering the growth rate in terms of cumulative deaths, it was less effective than the other NPIs.

### Travel restrictions

The decision to restrict travel was also taken throughout Europe during the same time period and not when cumulative cases and deaths were increasing by similar rates. However, there was no discrepancy in Rate E in terms of cumulative deaths since only France had recorded deaths 2 weeks before restricting travel. By a week later more countries were reporting deaths which were increasing by varying rates across Europe. This was possibly due to people having contracted the virus prior to the NPI being implemented.

The decision was likely taken at the start of the pandemic following the recommendations issued by the EU on March 16 2020, after the majority of countries had already cancelled events and closed schools [12]. In fact, prior to implementation the growth rates were lower than previously implemented interventions.

The travel restrictions were one of the most effective mitigation measures during the first wave in Europe as indicated by the consistently high improvements between growth rates, both in terms of cumulative cases and deaths. This is in agreement with existing studies such as those by Espinoza, et al. [11] and Primc and Slabe-Erker [26] which found that travel restrictions are highly effective at reducing transmission rates of COVID-19.

### Cancellation of events

Two weeks before events were cancelled, the virus had not yet reached all of Europe and cases were still increasing at low rates. Within the next week, the majority of countries were affected and cases were increasing more rapidly at varying rates across Europe. Similarly to school closure and travel restrictions, the decision to cancel events was taken during the same time period and not when countries were in similar stages in terms of cases.

Given that it was first to be implemented and at a time when cases had just started rapidly increasing, only two countries had registered their first deaths 2 weeks before cancellation causing the high variability in Rate E. By the following week a few more countries were registering their first deaths which were increasing at low and similar rates.

Hunter et al. [20] & Garchitorena et al. [17] argued that the cancellation of events, most notably superspreader events, had a large effect on reducing transmission. The results obtained agree with these studies and show that over time the cancellation of events did lower the rate of growth. However, it was one of the least effective in comparison to the other NPIs, especially in terms of cumulative deaths. Our understanding is that cancellation of events act as a reducer in the probability of significant jumps in the spike rate (due to superspreader events) rather than a lowering of the growth rate per se.

### Restrictions on gatherings

The decision to restrict gatherings was taken when countries were in the same stage in terms of cumulative cases as they were increasing by similar rates across Europe rather than at a similar timepoint. On the other hand, cumulative deaths were increasing at varying rates, possibly due to the additional delay from exposure to potential death.

The pre and mid intervention rates (rates A, E, and F) were also observed to be relatively lower than other NPIs. This could be because the effects of previously implemented interventions were being reflected in this intervention’s growth rates.

Brauner et al. [3] explain the effects of restricting gatherings depend on the limit set by countries. The lower the limit for gatherings is, the higher would be the effect of the intervention. The results show that the intervention had a moderate effect on the rate of growth for both cases and deaths and had one of the worst improvements in rates in comparison to the other NPIs. This could potentially mean that the limit set for gatherings by the majority of countries was not sufficient or possibly this is a dilution of the effects of earlier NPIs.

### Partial lockdown

The discrepancy observed in growth rates for cumulative cases shows that the partial lockdown was implemented when cumulative cases were growing by similar rates (four-fold increase over 2 weeks), whereas cumulative deaths were increasing by higher (six-fold increase over 2 weeks) and varying rates^4^ across Europe.

Before the implementation of the partial lockdown cumulative cases were increasing at lower rates, whereas cumulative deaths were increasing more rapidly in comparison to the previously implemented interventions. This could explain why countries felt the need to implement more stringent measures to control transmission.

This suppression measure was not implemented as early as the other interventions due to the negative consequences it has on the economy and mental health of individuals as well as the difficulty in monitoring compliance by individuals.

The studies by Flaxman et al. [14] and Ferguson et al. [13] found that in Europe lockdowns would have the highest impact on transmission rates and that their implementation would reduce transmission significantly. The results obtained when comparing the growth rates both in terms of cumulative cases and deaths, coincided with both studies and showed that the partial lockdown consistently had either the highest or one of the highest improvements between growth rates.

### Full lockdown

The countries that decided to implement a full lockdown did so when they were in very similar situations in terms of cases and deaths. This was shown by the very low variability in growth rates A, E, and F for both cumulative cases and deaths. Prior to implementation, cumulative cases and deaths were increasing by much lower rates, however, some countries wanted to further control transmission.

This intervention was one of the least effective at lowering the growth rate in terms of cumulative cases. On the other hand, it was more effective at lowering the rate by which cumulative deaths were increasing.

### Limitations

There where various limitation to this research. Firstly, the NPIs were implemented very close to each other which makes it difficult to measure their singular effect. The implementation of NPIs is also affected by other factors whose effects cannot always be measured. These include testing capabilities; timing, method and severity of implementation; population demographics socio-economic conditions; and public response and awareness [4, 5].

For example, countries with a higher population density and older population are more susceptible to cases and deaths [22, 30, 37]. Outbreaks in high-risk environments such as long-term care facilities also affect growth rates. A report by the European Centre for Disease Prevention and Control showed that in some European countries, 50% of deaths were attributable to patients in these facilities [9, 32]. Moreover these may have been unreported [25]. A similar discrepancy might have been that some countries may have had more time to report deaths while having Covid-19 as compared to deaths due to Covid-19. It would therefore be of great interest to revisit this work using excess mortality rates.

The growth rates could have decreased over time on their own after reaching a peak [38]. There were also missing values in the calculation of growth rates since not all countries had all rates and not all countries implemented all the interventions. Finally, the results obtained did not always apply to every country, that is, while the average rates could have shown a decrease from one rate to another, this might not have been the case for all countries.^5^

## Conclusion

For each NPI, the rate at which cumulative cases and deaths were increasing before the implementation of each intervention decreased over time. Without these interventions, the rate of growth would have increased, resulting in many more cases and deaths across Europe.

Our analysis indicates that partial lockdowns were the most effective from the interventions during the first wave in Europe as it reduced both incidence of cases and deaths. Travel restrictions also lowered both the number of cases and deaths. School closures and full lockdowns were effective in reducing the number of cases and deaths respectively.

## Data Availability

The datasets used and/or analysed during the current study are available from the corresponding author on reasonable request.

## Appendix 1

Table 9

**Table 9:** Date of Implementation of NPIs for Europe

NPI	School Closures	International Travel Restrictions	Cancellation of Public Events	Restrictions on Public Gatherings	Stay at Home Restrictions	
Description	All levels	Ban on High Risk Areas / Total Border Closure	Required cancellations	Limiting outdoor/indoor gatherings	Partial Lockdown	Full Lockdown
**Country**						
**Albania**	10/03/2020	15/03/2020	10/03/2020	22/03/2020	23/03/2020	30/03/2020
**Andorra**	16/03/2020	17/03/2020	13/03/2020	–	17/03/2020	–
**Armenia**	16/03/2020	24/03/2020	16/03/2020	16/03/2020	24/03/2020	–
**Austria**	16/03/2020	11/03/2020	10/03/2020	16/03/2020	16/03/2020	–
**Azerbaijan**	01/03/2020	29/02/2020	14/03/2020	14/03/2020	14/03/2020	24/03/2020
**Belarus**	–	14/03/2020	14/03/2020	–	–	–
**Belgium**	13/03/2020	18/03/2020	13/03/2020	18/03/2020	18/03/2020	–
**Bosnia and Herzegovina**	12/03/2020	24/03/2020	12/03/2020	12/03/2020	–	20/03/2020
**Bulgaria**	13/03/2020	17/03/2020	13/03/2020	17/03/2020	20/03/2020	–
**Channel Islands**	23/03/2020	20/03/2020	18/03/2020	29/03/2020	29/03/2020	–
**Croatia**	16/03/2020	19/03/2020	19/03/2020	19/03/2020	19/03/2020	–
**Cyprus**	09/03/2020	15/03/2020	15/03/2020	15/03/2020	24/03/2020	–
**Czech Republic (Czechia)**	12/03/2020	16/03/2020	10/03/2020	12/03/2020	16/03/2020	–
**Denmark**	16/03/2020	14/03/2020	12/03/2020	18/03/2020	–	–
**Estonia**	12/03/2020	17/03/2020	12/03/2020	25/03/2020	30/03/2020	–
**Faroe Islands**	12/03/2020	14/03/2020	12/03/2020	17/03/2020	–	–
**Finland**	18/03/2020	19/03/2020	13/03/2020	16/03/2020	16/03/2020	–
**France**	16/03/2020	18/03/2020	29/02/2020	16/03/2020	17/03/2020	–
**Georgia**	16/03/2020	21/03/2020	12/03/2020	23/03/2020	31/03/2020	–
**Germany**	16/03/2020	16/03/2020	10/03/2020	14/03/2020	21/03/2020	–
**Gibraltar**	23/03/2020	20/03/2020	17/03/2020	24/03/2020	24/03/2020	–
**Greece**	11/03/2020	23/03/2020	13/03/2020	18/03/2020	23/03/2020	–
**Hungary**	16/03/2020	17/03/2020	11/03/2020	11/03/2020	17/03/2020	04/05/2020
**Iceland**	16/03/2020	20/03/2020	16/03/2020	16/03/2020	–	–
**Ireland**	13/03/2020	24/03/2020	12/03/2020	12/03/2020	28/03/2020	–
**Isle of Man**	23/03/2020	23/03/2020	16/03/2020	25/03/2020	26/03/2020	–
**Italy**	23/02/2020	23/02/2020	23/02/2020	23/02/2020	23/02/2020	23/03/2020
**Kosovo**	12/03/2020	13/03/2020	11/03/2020	11/03/2020	13/03/2020	–
**Latvia**	17/03/2020	17/03/2020	14/03/2020	14/03/2020	17/03/2020	–
**Liechtenstein**	16/03/2020	18/03/2020	28/02/2020	16/03/2020	16/03/2020	–
**Lithuania**	16/03/2020	16/03/2020	12/03/2020	13/03/2020	16/03/2020	–
**Luxembourg**	16/03/2020	17/03/2020	13/03/2020	13/03/2020	17/03/2020	–
**Malta**	13/03/2020	20/03/2020	12/03/2020	28/03/2020	28/03/2020	–
**Monaco**	13/03/2020	11/03/2020	15/03/2020	11/03/2020	15/03/2020	–
**Montenegro**	16/03/2020	16/03/2020	16/03/2020	16/03/2020	30/03/2020	–
**Netherlands**	16/03/2020	17/03/2020	12/03/2020	12/03/2020	16/03/2020	–
**North Macedonia**	10/03/2020	18/03/2020	13/03/2020	13/03/2020	22/03/2020	–
**Norway**	12/03/2020	15/03/2020	12/03/2020	12/03/2020	12/03/2020	–
**Poland**	12/03/2020	15/03/2020	12/03/2020	24/03/2020	24/03/2020	–
**Portugal**	16/03/2020	12/03/2020	12/03/2020	12/03/2020	19/03/2020	–
**Republic of Moldova**	10/03/2020	17/03/2020	10/03/2020	10/03/2020	17/03/2020	–
**Romania**	11/03/2020	22/03/2020	06/03/2020	11/03/2020	22/03/2020	–
**Russian Federation**	16/03/2020	16/03/2020	10/03/2020	16/03/2020	28/03/2020	–
**San Marino**	09/03/2020	09/03/2020	09/03/2020	09/03/2020	14/03/2020	–
**Serbia**	16/03/2020	15/03/2020	02/04/2020	02/04/2020	02/04/2020	26/04/2020
**Slovakia**	12/03/2020	12/03/2020	12/03/2020	12/03/2020	08/04/2020	–
**Slovenia**	16/03/2020	28/03/2020	10/03/2020	10/03/2020	19/03/2020	–
**Spain**	09/03/2020	15/03/2020	03/03/2020	16/03/2020	16/03/2020	28/03/2020
**Sweden**	17/03/2020	19/03/2020	12/03/2020	29/03/2020	–	–
**Switzerland**	16/03/2020	16/03/2020	28/02/2020	13/03/2020	16/03/2020	–
**Turkey**	16/03/2020	27/03/2020	16/03/2020	16/03/2020	18/03/2020	–
**Ukraine**	11/03/2020	16/03/2020	11/03/2020	06/04/2020	15/03/2020	–
**United Kingdom**	20/03/2020	–	23/03/2020	23/03/2020	24/03/2020	–
**Vatican City**	n/a	n/a	08/03/2020	n/a	n/a	n/a

## Appendix 2

Table 10

**Table 10:** Mean Growth Rates in terms of Cumulative Cases

Mean Rate	School Closures	Travel Restrictions	Cancellation of Events	Restrictions on Gatherings	Partial Lockdown	Full Lockdown
**A**	603%	534%	627%	451%	410%	179%
**B**	525%	338%	558%	380%	247%	95%
**C**	171%	136%	222%	156%	120%	46%
**D**	91%	78%	125%	77%	57%	28%
**E**	5618%	4237%	3759%	2780%	3120%	499%
**F**	3519%	2544%	4137%	2168%	1600%	444%
**G**	1567%	909%	1909%	1110%	654%	184%
**H**	418%	319%	625%	353%	245%	87%
**I**	195%	157%	278%	155%	110%	49%

## Appendix 3

Table 11

**Table 11:** Mean Growth Rates in terms of Cumulative Deaths

Mean Rate	School Closures	Travel Restrictions	Cancellation of Events	Restrictions on Gatherings	Partial Lockdown	Full Lockdown
**A**	454%	562%	252%	494%	582%	160%
**B**	716%	408%	439%	424%	405%	96%
**C**	362%	315%	376%	286%	231%	62%
**D**	183%	157%	241%	157%	118%	33%
**E**	6250%	4275%	1597%	3356%	4988%	636%
**F**	3273%	3593%	1581%	2779%	2926%	340%
**G**	3205%	1560%	2209%	1624%	1404%	156%
**H**	1131%	967%	1473%	837%	640%	115%
**I**	536%	355%	719%	402%	275%	55%

## Appendix 4

Table 12

**Table 12:** Number of days between implementation of the cancellation of events NPI and the other five NPIs

	Number of Days between implementation of the cancellation of events NPI and the other five NPIs				
Country	School Closures	Travel Restrictions	Restrictions on Gatherings	Partial Lockdown	Full Lockdown
**Albania**	0.00	5.00	12.00	13.00	20.00
**Andorra**	3.00	4.00	–	4.00	–
**Armenia**	0.00	8.00	0.00	8.00	–
**Austria**	6.00	1.00	6.00	6.00	–
**Azerbaijan**	−13.00	−14.00	0.00	0.00	10.00
**Belarus**	–	0.00	–	–	–
**Belgium**	0.00	5.00	5.00	5.00	–
**Bosnia and Herzegovina**	0.00	12.00	0.00	–	8.00
**Bulgaria**	0.00	4.00	4.00	7.00	–
**Channel Islands**	5.00	2.00	11.00	11.00	–
**Croatia**	−3.00	0.00	0.00	0.00	–
**Cyprus**	−6.00	0.00	0.00	9.00	–
**Czech Republic (Czechia)**	2.00	6.00	2.00	6.00	–
**Denmark**	4.00	2.00	6.00	–	–
**Estonia**	0.00	5.00	13.00	18.00	–
**Faroe Islands**	0.00	2.00	5.00	–	–
**Finland**	5.00	6.00	3.00	3.00	–
**France**	16.00	18.00	16.00	17.00	–
**Georgia**	4.00	9.00	11.00	19.00	–
**Germany**	6.00	6.00	4.00	11.00	–
**Gibraltar**	6.00	3.00	7.00	7.00	–
**Greece**	−2.00	10.00	5.00	10.00	–
**Hungary**	5.00	6.00	0.00	6.00	54.00
**Iceland**	0.00	4.00	0.00	–	–
**Ireland**	1.00	12.00	0.00	16.00	–
**Isle of Man**	7.00	7.00	9.00	10.00	–
**Italy**	0.00	0.00	0.00	0.00	29.00
**Kosovo**	1.00	2.00	0.00	2.00	–
**Latvia**	3.00	3.00	0.00	3.00	–
**Liechtenstein**	17.00	19.00	17.00	17.00	–
**Lithuania**	4.00	4.00	1.00	4.00	–
**Luxembourg**	3.00	4.00	0.00	4.00	–
**Malta**	1.00	8.00	16.00	16.00	–
**Monaco**	−2.00	−4.00	− 4.00	0.00	–
**Montenegro**	0.00	0.00	0.00	14.00	–
**Netherlands**	4.00	5.00	0.00	4.00	–
**North Macedonia**	−3.00	5.00	0.00	9.00	–
**Norway**	0.00	3.00	0.00	0.00	–
**Poland**	0.00	3.00	12.00	12.00	–
**Portugal**	4.00	0.00	0.00	7.00	–
**Republic of Moldova**	0.00	7.00	0.00	7.00	–
**Romania**	5.00	16.00	5.00	16.00	–
**Russian Federation**	6.00	6.00	6.00	18.00	–
**San Marino**	0.00	0.00	0.00	5.00	–
**Serbia**	−17.00	−18.00	0.00	0.00	24.00
**Slovakia**	0.00	0.00	0.00	27.00	–
**Slovenia**	6.00	18.00	0.00	9.00	–
**Spain**	6.00	12.00	13.00	13.00	25.00
**Sweden**	5.00	7.00	17.00	–	–
**Switzerland**	17.00	17.00	14.00	17.00	–
**Turkey**	0.00	11.00	0.00	2.00	–
**Ukraine**	0.00	5.00	26.00	4.00	–
**United Kingdom**	−3.00	–	0.00	1.00	–
**Vatican City**	–	–	–	–	–

## Abbreviations

ACAPS: The Assessment Capacities Project
ECDC: European Centre for Disease Prevention and Control
NPI: Non-pharmaceutical intervention
WHO: World Health Organisation

## Footnotes

1 NPIs throughout the European continent have been analysed. A list of countries is provided in the Appendix.

2 ACAPS is an independent information provider providing humanitarian needs analysis and assessment [1].

3 $$\frac{5221}{2320}\div \frac{609}{100}\times 100=36.94\%$$.

4 Interquartile ranges of Rates A, E and F were 454, 4967 and 4375% respectively)

5 Only 7.4% and 6.2% showed an increase in weekly and fortnightly cumulative cases post NPIs.
